# Phenolic Compounds in Calafate Berries Encapsulated by Spray Drying: Neuroprotection Potential into the Ingredient

**DOI:** 10.3390/antiox10111830

**Published:** 2021-11-18

**Authors:** María E. Romero-Román, Mauricio Schoebitz, Jorge Fuentealba, Cristina García-Viguera, María D. López Belchí

**Affiliations:** 1 Departamento de Producción Vegetal, Facultad de Agronomía, Universidad de Concepción, Vicente Méndez #595, Chillán 3812120, Chile; mariaeugeromero@udec.cl; 2 Departamento de Suelos y Recursos Naturales, Facultad de Agronomía, Universidad de Concepción, Víctor Lamas 1290, Concepción 4030000, Chile; mschoebitz@udec.cl; 3 Departamento de Fisiología, Facultad de Ciencias Biológicas, Universidad de Concepción, Víctor Lamas 1290, Concepción 4030000, Chile; jorgefuentealba@udec.cl; 4 Phytochemistry & Healthy Foods Lab (LabFAS), Department Food Science & Technology, CEBAS-CSIC, Campus de Espinardo 25, Espinardo, 30100 Murcia, Spain; cgviguera@cebas.csic.es

**Keywords:** anthocyanins, *Berberis microphylla*, encapsulation, next generation ingredients, PC12 cells

## Abstract

Calafate is a berry rich in anthocyanins that presents higher content of polyphenols than other fruits. Its compounds have been described previously, however, the potential thereof in preventing and treating degenerative disorders has not yet been studied. Due to its astringency, the consumption of this berry in its natural state is limited. To profit from the aforementioned properties and reduce palatability issues, calafate berry extracts were microencapsulated by spray drying, a rapid, cost-effective and scalable process, and were then compared with freeze drying as a control. The stability of its contents and its in-vitro potential, with respect to AChE activity and neuroprotection, were measured from the obtained microcapsules, resulting from temperature treatments and different encapsulant contents. The results indicated that the spray-dried powders were stable, despite high temperatures, and their encapsulation exhibited nearly 50% efficiency. The highest quantity of polyphenols and 3-*O*-glycosylated anthocyanins was obtained from encapsulation with 20% maltodextrin, at 120 °C. Temperature did not affect the microcapsules’ biological action, as demonstrated by their antioxidant activities. The prevention of Aβ peptide cytotoxicity in PC12 cells (20%) revealed that encapsulated calafate can confer neuroprotection. We conclude that spray-drying is an appropriate technique for scaling-up and producing new value-added calafate formulations with anti-neurodegenerative effects and vivid colors.

## 1. Introduction

A Native Chilean berry, calafate (*Berberis microphylla* G. Forst), has been studied in the last decade due to its content of bioactive compounds. This fruit is an intensely purple berry with high anthocyanin content. It has been determined that calafate possesses 18 anthocyanins, derived from glycosylated delphinidin, petunidin, malvidin, peonidin, and cyanidin as well as some flavonols (quercetin, isorhamnetin, and myricetin) [1,2,3]. Anthocyanins’ importance lies in their benefits against chronic diseases and as a preventive treatment of degenerative disorders [4]. A recent study on calafate attributed this wild plant’s special desirable qualities for nutraceutical and industrial purposes to its phenolic compounds [1]. In addition, calafate and maqui, two Chilean berries, demonstrated regulated degradation of the extracellular matrix by the modulation of metalloproinases, and improvement in otherwise-impaired glucose uptake induced by a pro-inflammatory treatment [5]. Nonetheless, the potential of calafate’s phenolic compounds can still be explored.

Meanwhile, in the market, the consumer interest in products based on exotic fruits and native berries as health promoters has increased. Thus, the antioxidant and antibacterial capacity of natural compounds present in fruits have also been studied and fostered [6]. In these prepared plant-originating products, due to physical changes (light exposure, oxygen, and pH), the quality, color, taste, and benefits of the bioactive compounds can be diminished [7]. This hampers the use of these natural compounds as food pigments [8] and it can also limit anthocyanins’ efficacy when they are used for biological purposes. In order to solve the above-mentioned problems, techniques such as encapsulation have been utilized. The most commonly used techniques in the food industry are spray and freeze drying. Both procedures preserve the bioactive compounds for industrial purposes, bringing stability during storage as well as conferring resistance to pH changes along the intestinal tract [9]. The spray-drying process is usually used with a wall material that increases dry matter, immobilizes and preserves the target sample (fruit extract), while freeze-drying often uses whole berries or pieces of fruits. The advantage of these techniques is not only at an industrial level, but also confers stability to bioactives after ingestion, so they can increase or maintain their functionality.

Spray-drying is widely used in the large-scale production of encapsulated formulations, being economical and adaptable and producing an excellent quality of product. It can bring protection and stability to bioactive compounds, providing a physical barrier as well as revealing flavors. The process involves high temperatures in different spots of the equipment. In fact, as high is the inlet air temperature as dry is the final product. Another crucial point in this process is the air:solution ratio, with consideration to humidity and solubility, and dispersibility of the final samples [10]. All the steps mentioned above, in this technique, are inconvenient in terms of the loss of bioactive content; therefore, the process must be optimized [11].

On the other hand, freeze drying is a process that isolates the water of the samples by freezing and subsequent sublimation that is carried out in a vacuum and at a low temperature. It is understated as a process for drying products without altering their qualitative or quantitative compositions [12]. Nonetheless, freeze-drying is a slow process, and the production cost is elevated, and, therefore, despite the drawbacks of working at high temperature, spray drying has several advantages to consider, such as industrial scalability and a faster process as compared with freeze drying. In addition, maltodextrin is a polymer widely used in spray drying to immobilize as well encapsulate molecules used in the food industry and for medical purposes [13].

Studies with berries have demonstrated the effectiveness of both mentioned techniques in developing formulations that protect bioactive compounds for natural colorants, conferring improved stability, solubility, dispersibility, and bioavailability [14]. In cancer studies, bilberry formulations were used to exhibit the anticancer activity of anthocyanins and, after spray drying, the chemo-preventive bioactivity of bilberry anthocyanins was shown to have remained [15]. In addition, it has been shown that formulations using polyphenols from berries can help to cross the blood-brain barrier and have activity at the central nervous system [16]. Recently it has been proven that fruits rich in antioxidants could prevent and counteract neurodegenerative processes, providing functional benefits against cellular alterations observed in Alzheimer’s disease [17]. The inhibition of acetylcholinesterase enzyme (AChE) activity is an indicator of neurotransmitter dysfunction in diseases such as Parkinson and Alzheimer’s disease confirmed by studies with microcapsules from Hippophaë rhamnoides determined correlations between its compounds and in-vitro anticholinergic and the antioxidant activity [18]. There is no evidence of calafate’s compounds related to anti-neurodegeneration and no previous works in terms of the encapsulation of calafate. This work aims to examine novel encapsulated formulations based on calafate berries maintaining their high antioxidant capacity, using spray-drying techniques in order to preserve anthocyanins from degradation. Accordingly, calafate powders and the influence of spray-drying conditions on the physicochemical, antioxidant properties, and biological activity through enzymatic and cellular assays were studied to design a next-generation ingredient to be used in the food industry for nutrition, health, and wellness purposes.

## 2. Materials and Methods

### 2.1. Material

Ripe berries were collected in Coyhaique, Aysén Region, (45°33′18″ S, 71°50′38.399″ W) for two consecutive years (2018, 2019). They were frozen and transported to the Laboratory of Chemical Analysis (Department of Plant Production, Faculty of Agronomy, University of Concepción, Concepción, Chile) and kept at −80 °C until processed and analyzed.

### 2.2. Chemicals

Maltodextrin DE-10, as a carrier agent, was purchased from Sigma-Aldrich Chemical Co. (St. Louis, MO, USA). Analytical grade reagents such as formic acid, acetonitrile, methanol, water, monobasic sodium phosphate, dibasic sodium phosphate, and ethanol were acquired from Merck (Darmstadt, Germany). Commercial standards of delphinidin 3-glucoside (95%), cyanidin 3-glucoside (98%) chlorogenic acid, rutin trihydrate, quercetin dihydrate, radical 2,2-diphenyl-1-picrylhydracil (DPPH*), Trolox, fluorescein (free acid), and 2,2-azobis-(-2-methylpropionamidine) dihydrochloride (AAPH) were acquired from Sigma Aldrich (St. Louis, MO, USA). For the enzymatic assays, *E. electricus* AChE, acetylthiocholine iodide (ATCh), and 5,5′-dithio bis(2 nitrobenzoic) acid (DTNB) were supplied by Sigma-Aldrich Chemical Co. (St. Louis, MO, USA).

### 2.3. Preparation of Microcapsules by Spray Drying and Study of Storage Stability

The mashed calafate berries (100 g) were mixed with ethanol 96% (*w*/*v*), food grade, in 1:6 (*w*/*v*) and stirred for 24 h at room temperature. After that, alcohol was removed using a vacuum pump. Next, calafate blend was mixed with maltodextrin (MD) (10, 20, and 30 g) and stirred for 12 h in distilled water. The resulting solutions were homogenized by constant agitation while they were fed into a mini spray-dryer B-290 (Büchi, Flawil, Switzerland) at room temperature. The spray-dryer was operated at an air inlet ranging from 100, 120, 140 ± 3 °C. Therefore, nine treatments were carried out considering the three doses of maltodextrin mentioned above and three different inlet temperatures. The rate of feeding and airflow was constant (1 mL min^−1^ and 600 L h^−1^ respectively). The resulting powders were collected in amber glass bottles and stored at 5 and 35 °C.

Otherwise, whole calafate berries were freeze-dried with an OPERON model OPR-20160701-1A1EO (Gyeonggi-do, Korea) freeze dryer, under a pressure below 0.05 mBar, −70 ± 2 °C for 48–72 h, and stored at −80 °C until analyzed [1]. This process was used as the most suitable control process to protect bioactive compounds.

Finally, powder recovery (ratio between the quantities of powder versus the initial mass solids), and entrapment efficiency (g of phenolic compounds from calafate extracts encapsulated 100 g^−1^ phenolic compounds from calafate extracts added) were calculated.

### 2.4. Characterization of Phenolic Compounds and Antioxidant Properties from Encapsulated Calafate

The chemical characterization of the microencapsulated and freeze-dried fruits was performed with an High Performance Liquid Chromatography-Diode Array Detection-Electrospray Ionization/Mass Spectrometry (HPLC-DAD-ESI/MSn) system. The antioxidant capacity was determined by spectrophotometry 2,2-diphenil-1-picrilhidracilo (DPPH*) and oxygen radical absorbance capacity (ORAC) assays. In addition, the size powder, yield, recovery, and stability of the capsules were also measured.

The chemical analyses were conducted under the following protocol [19]. Briefly, 50 mg of each sample (encapsulated or freeze-dried), were first poured into 1.5 mL of 25:24:1 (methanol:water:formic acid) stirred for 5 min, and centrifuged at 10,000× *g* rev min^−1^ for 10 min. Next, the tubes were submerged in ultrasound bath (1 h) and kept at 4 °C overnight. Then, the samples were filtered through a 0.22-μm PVDF membrane (Millex V13, Millipore, Bedford, MA, USA) and put in amber vials for chromatographic analyses. Chromatographic analyses were performed in an Agilent Technologies 1220 Infinity Liquid Chromatograph equipped with an autoinjector (G1313, Agilent Technologies, Santa Clara, CA, USA) coupled with a diode array detector (1260, Agilent Technologies, Santa Clara, CA, USA) and a Luna 5-μm C18, 100-Å column (250–4.6 mm) and security guard cartridges PFD C18 (4–3.0 mm) (Phenomenex, Torrance, CA, USA). Mobile phases were 1% formic acid (A) and methanol (B), and a flow rate of 0.9 mL min^−1^. The analyses procedure was based on calibration curves using the following standards: cyanidin 3-O-glucoside at 520 nm for anthocyanins, quercetin 3-O-rutinoside at 360 nm for flavonols, and cinnamic acid derivatives as 3-O-caffeoylquinic acid at 320 nm. All were expressed in mg g^−1^.

The DPPH analysis was done measuring the variation in absorbance at 515 nm after 30 min of reaction with the radical DPPH* [20], using Thermo Scientific UV-Vis Orion AquaMate 8000 spectrophotometer (Madrid, Spain). Six replications were carried out per sample. The results were expressed in µmol Trolox equivalent (TE) g^−1^ of sample dry weight (DW). An ORAC assay was performed according to a described methodology [21] using black-walled 96-microwell plates (Nunc, Roskilde, Denmark) and an Infinite^®^M200 microplate reader (Tecan, Grödig, Austria). Each well received 150 μL of fluorescein solution and 25 μL of phosphate buffer, Trolox^®^ solutions, or sample solution to measure the blank, the curve, or the samples, respectively. Samples were placed in the microplate reader; after 30 min of incubation (37 °C), AAPH solution (25 μL) was added to each well, and fluorescence was recorded every 5 min for 120 min, using an excitation wavelength of 485 nm and an emission wavelength of 520 nm. ORAC values were calculated using the difference in areas under the fluorescein curve between the blank and a sample. The results were expressed as µmol of Trolox g^−1^ of dry weight. Peroxyl radical was generated using 2,2′-azobis (2-amidino-propane) dihydrochloride and was prepared fresh for each run. Fluorescence conditions were as follows: excitation at 485 nm and emission at 520 nm.

### 2.5. Neuroprotective Properties of Calafate Microcapsules

The enzymatic assays of acetylcholinesterase (AChE) were performed using the Ellman method [22]. Different concentrations of acetylthiocholine were prepared with distilled water (1 mM, 0.5 mM, 0.2 mM, and 0.1 mM). The solution of chromogenic reagent was carried out by dissolving 0.16 g of DTNB and 0.6 g of NaHCO_3_ in 40 mL of 0.1 M phosphate buffer at a pH of 7.6. The AChE enzyme, from *E. electricus*, was dissolved in distilled water (5 U mL^−1^). The control assay contained 50 µL of enzyme, 200 µL of DTNB, 2.6 mL of 100-mM phosphate buffer (pH 7.6), and 200 µL of acetylthiocholine (1 mM). For solvent control, a mixture of ethanol/formic acid/distilled water (24:25:1) was prepared and 100 µL was added to the vials. The inhibition reaction was done with 100 µL of calafate powder extract at two dilutions (1/10 and 1/100 *w*/*v*). The spectrophotometric study was incubated at 37 °C for 15 min and the absorbance was measured with a Thermo Scientific UV-Vis Orion AquaMate 8000 spectrophotometer (Madrid, Spain) at 412 nm. The phosphate-buffer solution was used as a blank. Six replications were carried out.

#### 2.5.1. PC-12 Cells

Cell viability and cytotoxicity were determined by the use of powders to test the cell toxicity of PC12 cells. The cell viability assay MTT was carried out using polyphenols from calafate berry powders in a chronic treatment by triplicate for 24 h.

PC12 cells from ATCC (Manassas, VA, USA) were cultured in DMEM with 5% fetal bovine serum, 100 U/mL penicillin, 100 μg/mL streptomycin, and 2 mM l-glutamine. The cells were incubated under standard conditions (37 °C, 5% CO_2_) and when 80% confluence was achieved, the cells were treated with 0.25% trypsin for 10 min, washed and resuspended in HyQ DMEM/High-Glucose (Hyclone, Logan, UT, USA) with 5% fetal bovine serum (Hyclone), 2 mM l-glutamine (Gibco, Grand Island, NY, USA) and 1% penicillin-streptomycin (Gibco). The cells were then plated at a concentration of 50,000 cells/well for experiments and used 24 h after plating under experimental conditions similar for neurons.

#### 2.5.2. Soluble Oligomers of Aβ (SO-Aβ) Preparation

Amyloid beta (Aβ_1–40_) peptide (rPeptide, Bogart, GA, USA) was reconstituted in dimethyl sulfoside (DMSO) at a concentration of 2.3 mM. Then, 2 μL aliquots were dissolved in sterile distilled water, in order to reach an 80-μM concentration. To generate the oligomeric forms, the solution was subjected to vertical stirring (500 rpm) using a magnetic agitator, for 2 h at room temperature, similarly to previous works [23,24]. The presence of SO-Aβ was tested by Western blot (Figure S1). The aforementioned peptide solution, at a concentration of 0.5 μM, was used.

#### 2.5.3. Cell Viability Assay

In order to evaluate changes in cell viability by measuring the ability of mitochondria to reduce 3-[4,5-dimethylthiazol-2-yl]-2,5-diphenyl tetrazolium bromide (MTT salt) to formazan, an in-vitro MTT assay kit (Sigma-Aldrich, Saint Louis, MO, USA) was used. The cells, subjected to different experimental conditions, were incubated for 30 min in MTT (1 mg/mL); the insoluble formazan was solubilized in 100 μL of 2-propanol, and the absorbance was registered (560 nm and 620 nm) in a NOVOstar multiplate reader (BMG Labtech, Offenburg, Germany).

### 2.6. Scanning Electron Microscopy of Microcapsules

The morphology of the particles was evaluated by scanning electron microscopy (SEM). The microcapsules were attached to a double-sided adhesive tape mounted on SEM stub, coated with 3–5 mA gold/palladium under vacuum scanning electronic microscope (FEI—Inspect S50, FEI Company, Hillsboro, Oregon) working at 5 kV, with 10,003 and 50,003 magnification and operated in high-vacuum mode [25]. The particle mean size analysis was calculated by measuring 30 randomized particles in the microscope plate.

### 2.7. Statistical Analysis

From the results obtained, the experimental errors, randomness, normality (Shapiro test), and homogeneity of variances (Levene’s test) were analyzed. Once the assumptions were verified, the analysis of variances, the test of differences in means (Tukey), significance level *p* < 0.05 were performed. In the evaluation of the stability of the calafate anthocyanin powders, the analysis of variances, test of differences of means (Tukey), and the adjustment of regressions with a significance of 5% were carried out. These analyses were done in the software R by RStudio, version 1.4.1106 (PBC, Boston, MA, USA) [26].

## 3. Results

### 3.1. Polyphenols Recovery from Calafate Formulations

This study considered nine treatments of combinations of percentages of maltodextrin (three doses, 15, 20, and 30%) and three inlet temperatures of spray drying (100, 120, and 140 °C) to keep calafate polyphenols on maltodextrin (MD) and freeze drying as a control. First of all, the entrapment efficiency, recovery mean of particle sizes of all formulations and compared them, were calculated in terms of anthocyanins and other phenolic compounds.

The entrapment efficiency ranged from 41.10 to 56.72% and recovery reached 61.36%, similar to the means reported using a spray-drying technique to encapsulate sweet potatoes’ anthocyanins under varying inlet temperatures [27]. Neither the recovery of all treatments nor the size of the particles were significantly different. On average, the total anthocyanin contents (TACs) in spray drying ranged from 11.87 to 14.75 mg g^−1^ DW, with significant difference only with the control (freeze-dried) treatment (Table 1). Despite the reduction of phenolic bioactivity in spray-drying, a high anthocyanin content in the obtained microcapsules was demonstrated. The relation between TACs and total polyphenol contents (TPCs) is marked by the similarity in their quantifications in all treatments.

Before the encapsulation process, eight anthocyanins and eight flavonols were identified and quantified by HPLC-DAD-(ESI)/MSn in calafate berry extracts (Table 2). The values of quantification reflected the concentration of phenolic compounds in the calafate fruit (DW) at the starting point. The main anthocyanins of calafate berry extracts were delphinidin 3,5-dihexoside, and malvidin 3,5-dihexoside [2,28,29]. The three principal flavonols: quercetin 3-rutinoside, quercetin 3-glucoside, and isorhamnetin 3-O-hexoside-derivative had been also reported before in *B. microphylla* [1,29] and in a wild raspberry [30].

**Figure 1:**
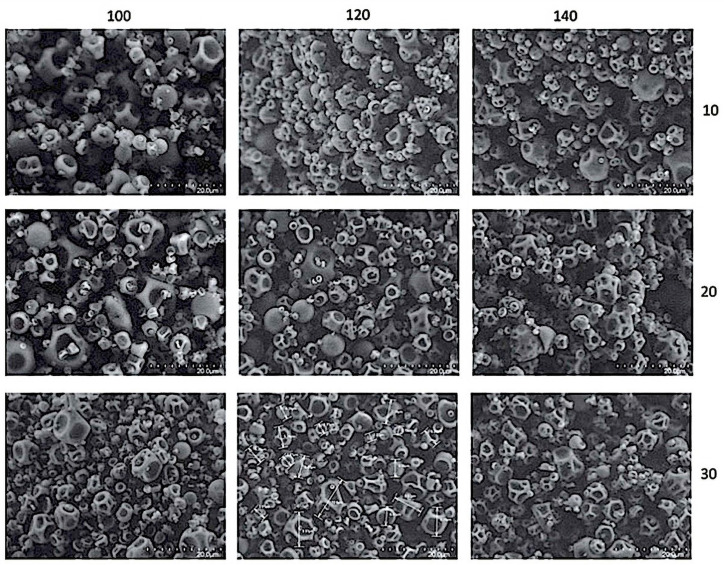
SEM images of spray-dried calafate berry powders. Three doses of maltodextrin and three inlet temperatures. Rows belong maltodextrin percentage (10, 20, 30) while columns correspond to inlet temperature of the spray dryer (100 °C, 120 °C, 140 °C).

Once the encapsulation was carried out, micro-photos from the formulations were visualized and analyzed (Figure 1). SEM micro-photos for the different treatments showed spherical forms and a majority of their surfaces were depressed. The differences in sizes responded to temperature treatments. The photos also confirmed that the maltodextrin used as a matrix to encapsulate the *B. microphylla* compounds, worked effectively and efficiently [12]. It was also possible to estimate the particle sizes of the calafate coatings rich in anthocyanins through this visualization. The size and agglomeration was related to the inlet temperature of the spray dryer, but, generally we could assert that formulations of T1 and T4 probably had not capped the bioactives of calafate effectively, due to the larger particle sizes found, in comparison with other treatments caused by the low temperature (100 °C). Due to agglomeration, some particles showed a slightly rough surface. Particle sizes, with respect to the temperatures used, were stable (average 7 µm).

### 3.2. Antioxidant Activity and Calafate Anthocyanin Stability at Different Temperatures of Storage

**Figure 2:**
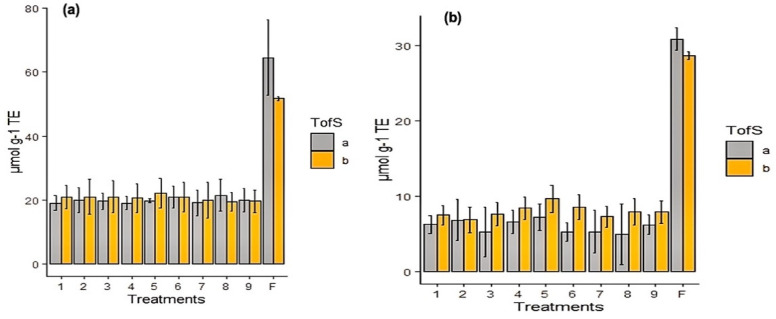
Antioxidant and enzymatic activity of encapsulated calafate at two storage temperature (TofS, temperature of storage a = 5 °C, b = 35 °C) 1–9 spray dried treatments, freeze-dried (control process). (**a**) DPPH assay: Comparison between nine formulations by spray-drying, with freeze-drying of calafate as a control process; (**b**) ORAC assay: Spray-drying formulation, with freeze-drying of calafate as a control process.

On the other hand, the 2,2-difenil-1-picrilhidracilo (DPPH) and oxygen radical absorbance capacity (ORAC) assays were defined using all nine spray-drying treatments and freeze-dried calafate as a control. The DPPH results achieved were in a range between 15.5 and 16.8 µmol g^−1^ TE for spray-drying treatments, and 51 to 57.9 µmol g^−1^ TE in the control (freeze-dried). The antioxidant activity was confirmed by ORAC assay and the means of treatments (from T1 until T9) was 14.55 µmol g^−1^ TE (Figure 2a,b). Slightly superior results were obtained with our formulations when compared to the spray-dried powders of sweet potatoes [27].

**Figure 3:**
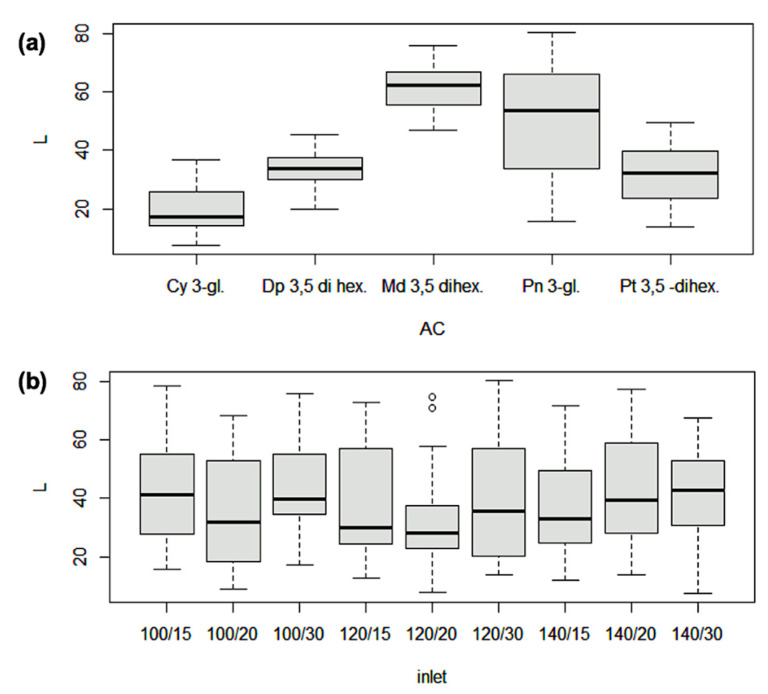
Anthocyanin losses (%) from spray dried microcapsules of calafate (L) in (**a**) individual anthocyanins; (**b**) comparison of total anthocyanin losses, considering all treatments (temperature/percentage of maltodextrin).

Regarding the storage temperatures of the calafate microcapsules, statistical differences in the losses of anthocyanins between them were found. Minor losses were revealed for cyanidin 3-glucoside at 35 °C (Figure 3a) of 17.4%, while the highest were for peonidin 3-glucoside and malvidin 3,5-dihexoside, of 58.2% and 67.3% respectively. Similarly, slight differences between the interaction of anthocyanins, maltodextrin, and the inlet temperature of spray drying shown that best treatment was the combination of 120 °C and 20% of maltodextrin for minimizing anthocyanin loss (Figure 3b).

The comparison of the main anthocyanins found, as a function of inlet temperature, were expressed in percentage (%) of loss (Figure 3b). The behavior of all anthocyanins seemed to be similar for all treatments. However, the range of loss was higher for peonidin 3-glucoside and lower for delphinidin 3,5-dihexoside. The losses reported in this research were consistent with results described (60% lost) for the microencapsulation of anthocyanins from blueberries [10].

Additionally, the variations in the main anthocyanins presenting in the microencapsulated calafate over time showed significative differences between all treatments when considering individual anthocyanins (Table 3). The treatment T2 presented an 11% loss of cyanidin 3-glucoside, while T6 lost 65.3% compared to the initial quantity of peonidin 3-glucoside. Some of the main anthocyanins were not detected after the 336 days, such as petunidin 3,5-dihexoside, peonidin 3-glucoside, and malvidin 3,5-dihexoside, especially in the assay of storage temperature (35 °C).

### 3.3. In-Vitro Study of Neuroprotective Properties of Calafate Microcapsules in Neurodegenerative Models

**Figure 4:**
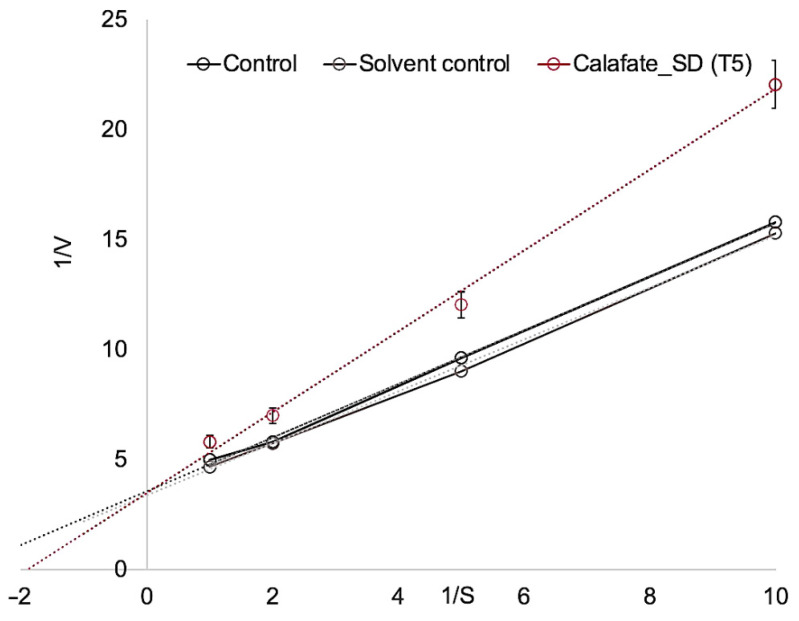
Acetylcholinesterase activity (AChE) of powder of calafate by Spray Drying.

To study the neurodegenerative properties, the best resulting spray-dried treatment in relation to bioactive content was used (T5 with 19.02 mg g^−1^ of polyphenols). This treatment presented the lowest difference with the control and consequently the greatest antioxidant capacity. The inhibition of acetylcholinesterase (AChE) was determined by the Ellman method [22] with the use of 100 μL of two calafate concentrations (1/10 and 1/100, *w*/*v*) in relation to the aforementioned treatment of encapsulated calafate; treatments at lower concentrations were excluded due to insignificant outcomes. The type of inhibition calafate powder exhibited against acetylcholinesterase was competitive, confirmed by the Lineweaver–Burk plot with kinetic inhibition (Figure 4). Calafate formulation showed the best inhibition, from 0.2 mM of substrate concentration *Ki* = 1.2872; ergo, it was more effective than the control. We note, however, that studies have reported that higher concentrations of polyphenols in enzymatic assays can easily saturate the medium and could overlap the inhibitor action [31].

The Calafate bioactives joined to the enzyme, preventing it from binding to the substrate; therefore the activity of the enzyme was diminished, suggesting that the calafate could enhance cholinergic tone and promote and restore acetylcholine (ACh) functionality. The AChE enzymatic inhibition of calafate encapsulated by spray drying showed slightly lower values with respect to those reported for the Chilean strawberry [32], and higher values in comparison with microcapsules from Tintorera grapes [25]. These differing results are due to the kinds of phenolic compounds and concentrations of polyphenols in berry extracts; strawberry and tintorera grapes are not entirely comparable, although in all cases the biological potential of phenolic compounds on the enzyme acetylcholinesterase was observed.

In addition, the neuroprotection was confirmed by the PC12 cells model assay. The cells pretreated with the amyloid beta peptide (0.5 µM, 24 h), a key toxic stimulus mimicking the main cellular toxicity in Alzheimer Disease, maintained their viability when microencapsulated calafate was co/incubated with the peptide. The effect of other components of the powders, such as maltodextrin, has been demonstrated as irrelevant in terms of biological activity, even in clinical trials [11].

**Figure 5:**
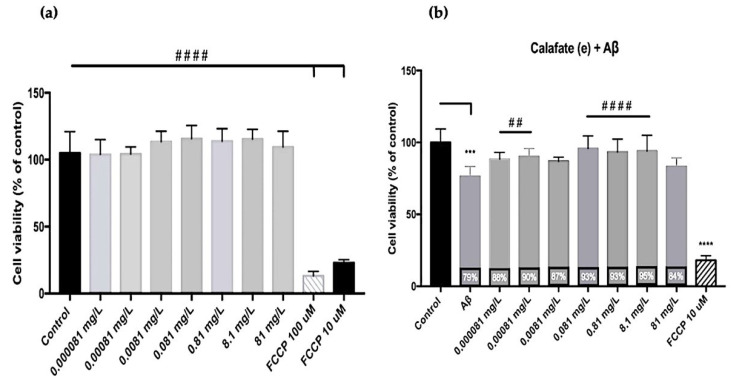
Effect of calafate powders on PC12 cell viability. (**a**) Comparative graphs of cell viability with increasing concentrations of the powder; (**b**) effect of encapsulated calafate on the reduction of cell viability induced by amyloid β-peptide oligomers (0.5 uM). Acetylcholinesterase activity (AChE) of powdered calafate, obtained by spray drying. FCCP: carbonyl cyanide4-(trifluoromethoxy)phenylhydrazone; Aβ: Amiloid beta. Statistical significance is found as *** (*p* < 0.01), ## (*p* < 0.05) and ****/#### (*p* < 0.001).

After the evaluation of the encapsulated calafate polyphenols, as we have mentioned, the treatment (T5) was used to determine the effect of microencapsulated calafate on cell-viability assays. Cell viability was not affected by the presence of the extract only, using different extract concentrations (8.1 × 10^−5^–81 mg L^−1^), suggesting their safe use in this cellular model. In these experimental approaches, carbonyl cyanide4-(trifluoromethoxy)phenylhydrazone (FCCP) (10–100 µM), a mitochondrial uncoupler that generates potent oxidative stress, was used as a positive control of toxicity, reducing cell viability by about 90% (Figure 5a). Values are presented as percentages with respect to the control and with respect to Aβ (#) (*n* = 3). Calafate did not reduce the viability of PC12 cells, suggesting that the activity of encapsulated calafate, here, could be similar to that reported for maqui berry extracts [33]. In the same range of concentrations evaluated above, PC-12 cells exposed to Aβ peptide demonstrated a reduced viability of approximately 20% with respect to control conditions, while co-incubation with encapsulated calafate induced viability recovery by preventing the cytotoxicity of Aβ peptide in a concentration-dependent manner (Figure 5b). Lower concentrations of used calafate (0.000081 and 0.00081 mg L^−1^) restored viability of the Aβ toxic effect by 10%. At higher concentrations (0.081–8.1 mg L^−1^) the viability of the toxic effect of the peptide was restored to about 20%. A report using quercetin (one of the compounds reported in encapsulated calafate) on a PC12 cell model showed the positive influence of this compound in increasing the survival rate of PC12 and reduced the toxicity of Aβ [16], as we have demonstrated by the use of encapsulated calafate.

## 4. Discussion

The nutritional relevance of non-traditional fruits is characterized by a plethora of vitamins, nonessential nutrients, and bioactive compounds. This last group of compounds can be obtained not only from fruits but also from many vegetable-origin products that confer important health-beneficial activities, such as antioxidant, antibacterial, and anti-inflammatory activities, among others [34]. In this research we highlighted the antioxidant activity as well as the neuroprotection potential of calafate bioactive compounds and the efficacy of its encapsulation to protect them and ensure the retention of their claimed health benefits.

In the physical characterization of calafate formulations obtained by spray drying, this paper addressed the influence of temperature on particle size, entrapment efficiency, and recovery. Slight differences between all spray-drying treatments compared to freeze drying (FD) were found. The results were statistically different, considering freeze drying concentrates fruit content by removing water, while, in spray drying, the compounds were subjected to high temperatures that compromise calafate bioactives’ concentrations. Despite of the aforementioned facts, no essential losses of bioactive compounds were observed, demonstrating that spray drying would be an adequate technique to carry out the drying process of calafate berries. Capping theloss of bioactives, together with the advantages of management on a continuous basis, low operating cost, the high quality of capsules in good yield (rapid solubility, small size, and high stability), demonstrate the usefulness of the spray-drying process. Some authors have suggested wider ranges of temperatures for reaching particles of uniform size and greater efficiency [27]. In our case, higher temperatures lowered the size of the calafate microencapsulated particles, as observed by electronic microscopy.

Regarding total polyphenol and total anthocyanins content, the spray-dried formulations did not show statistical differences. Our findings appear to be well substantiated if we consider that the range of inlet temperature was not as wide as in other studies. Consequently, the use of 100–140 °C considerably reduced the losses of anthocyanins as compared with other encapsulation studies [10,35]. Given that calafate formulations, rich in polyphenols, have been shown to be stable, minor loses were nonetheless found in individual anthocyanins over time (20–30%) in the spray-dried treatments. Studies of anthocyanins’ stability have confirmed similar losses due to the temperature of storage that justify the observed degradation, as well as some of the changes in anthocyanins’ structures [12]. It is worth noting the high content of these bioactive compounds found in calafate berries, and that, irrespective of storage temperature, calafate formulations maintained adequate content of glycosylated delphinidin, cyanidin, and malvidin.

The main metabolites studied and analyzed in microcapsules of calafate have already been characterized before in extracts of this fruit (delphinidin 3,5-dihexoside, and malvidin 3,5-dihexoside quercetin 3-rutinoside, quercetin 3-glucoside, and isorhamnetin 3-O-hexoside-derivative) [1,2]. These compounds, found in other fruits, have also been associated with beneficial effects on human health [36,37] and have been used to assert their potential in coping with neurodegenerative disorders [16].

Plant natural products have shown to be a promising source of acetylcholinesterase inhibitors. In fact, two of the drugs approved for Alzheimer’s disease are galantamine and rivastigmine, two alkaloids derived from plants [38]. Although, our findings were responsive in in-vitro assays and experimental tests; from this research, we can recommend calafate formulations as a palliative treatment due to their nature and demonstrated inhibition of AChE. Considering calafate powder as a competitive inhibitor for binding AChE enzymes reversibly at the same site as the acetylcholine, as demonstrated by the Michaelis–Menten constant (*Km*), the use of calafate formulations is crucial in understanding how useful this berry can be in terms of palliating neurodegenerative disorders. The obtained DPPH* and ORAC assays results effectively estimated calafate’s antioxidant capacity and show that its active redox properties are complementary to the neuroprotection described in the neurotoxicity assays (Figure 5) due to polyphenols attenuating toxicity, modulating lipid peroxidation [39], and protecting against oxidative stress-induced neurotoxicity [40]. Nonetheless, it is necessary to contrast these findings with in-vivo tests to clarify the true role of these metabolites in our health; however, these results appear promising. Encapsulated calafate, rich in anthocyanins, would not induce side effects and could be a dietary alternative to be included in the food industry. The microencapsulation of calafate also represents a strategy by which it could act as a carrier of the bioactive compounds present in the extract, towards the central nervous system, given the difficultly of access due to the blood–brain barrier.

It has been demonstrated that the polyphenols present in berries are capable of improving cognitive properties and prevent or reduce the risks of neurodegenerative diseases [17]. In addition to this study, the potential of calafate berries and of encapsulated calafate for preventive treatments of neurodegenerative disorders can be clarified by the use of PC12 cells, advancing the study of their neurotoxic activities as a model for neurodegenerative diseases.

## 5. Conclusions

In terms of encapsulation, there had been no evidence of encapsulated calafate improving the protection and stability of its compounds until now. The main potential of calafate is found in its great anthocyanin content, therefore, using typical processes such as spray drying to develop new ingredients for the food industry marks a valuable contribution. Similarly, the enzymatic activity (acetylcholinesterase) and the influence of calafate in PC12 cells to determine calafate microcapsules’ potential as an alternative for neurodegenerative diseases had not been yet studied.

Encapsulation and protection for calafate berries by spray drying was effective in terms of preserving its polyphenol content, antioxidant capacity, and inhibition of AChE enzymes, as well as its recovery viability by preventing the cytotoxicity of Aβ peptide in PC12 cells related to neurodegenerative disorders. In the temperature range of the process studied, no differences were observed in relation to antioxidant capacity and hence biological potential, showing, in all cases, a high anthocyanin content. The study of storage temperatures demonstrated that the encapsulation strategy for calafate is effective in preserving and protecting bioactive compounds for a longer period of time. The spray-drying technique can be used for calafate berries in order to scale-up and produce a next-generation ingredient with anti-neurodegenerative potential.

## Figures and Tables

**Table 1:** Entrapment efficiency (EE), recovery, size of microcapsules, and quantification of phenolic compounds of different dried treatments.

Code	Treatment	EE (%)	Recovery (%)	Size (µm)	TACs (mg g^−1^)	TPCs (mg g^−1^)
	(MD-Temp)					
T1	15–100	43 ± 0.48 ^a^	56.9 ± 1.26 ^a^	7.51 ± 2.9 ^a^	12.6 ± 0.87 ^a^	16.6 ± 0.7 ^a^
T2	15–120	50.4 ± 0.83 ^a^	59.3 ± 3.4 ^a^	8.05 ± 5.85 ^a^	12.2 ± 1.23 ^a^	15.4 ± 1 ^a^
T3	15–140	49.2 ± 1.2 ^a^	57 ± 2.5 ^a^	7.07 ± 4.78 ^a^	12.2 ± 1.15 ^a^	15.4 ± 0.9 ^a^
T4	20–100	41.1 ± 1.99 ^a^	58 ± 3.2 ^a^	6.69 ± 3.32 ^a^	13 ± 2.4 ^a^	16.5 ± 1.5 ^a^
T5	20–120	46.3 ± 2.27 ^a^	58.3 ± 1.8 ^a^	7.64 ± 4.63 ^a^	14.8 ± 1.02 ^a^	19 ± 0.8 ^a^
T6	20–140	42.4 ± 2.16 ^a^	60.1 ± 4.2 ^a^	6.63 ± 3.66 ^a^	12.5 ± 0.67 ^a^	15.9 ± 0.6 ^a^
T7	30–100	46.3 ± 2.43 ^a^	59.5 ± 1.87 ^a^	6.89 ± 3.29 ^a^	13.2 ± 0.34 ^a^	16.5 ± 0.3 ^a^
T8	30–120	56.7 ± 0.5 ^a^	58 ± 2.21 ^a^	6.5 ± 3.19 ^a^	13.1 ± 1.02 ^a^	16.5 ± 0.8 ^a^
T9	30–140	47.2 ± 0.56 ^a^	61.4 ± 3.87 ^a^	6.61 ± 3.7 ^a^	11.9 ± 2.1 ^a^	14.9 ± 1.2 ^a^
F	Freeze-Drying	82.5 ± 5.6 ^b^	93.7 ± 2.5 ^b^	-	20 ± 3.8 ^b^	24.8 ± 2 ^b^

The treatments consisted of combinations of three doses of maltodextrin (MD) (15, 20, 30%) and three inlet temperatures (Temp) of the spray-dryer (100, 120 and 140 °C). TACs—total anthocyanins, expressed in mg g^−1^ of dried weight (DW); TPCs—total phenolic compounds, expressed in mg g^−^^1^ of dried weight. Different letters in the same column mean significant differences at (*p* ≤ 0.05). MD-Temp: Maltodextrin-Temperature; EE: entrapment efficiency.

**Table 2:** Phenolic compounds in calafate fruit extract (Berberis microphylla G. Forst) identified and quantified by HPLC-DAD-(ESI)/MSn.

Retention Time	λ (nm)	M+ or M−	Ion	MSn	Main Phenolic Compounds	Concentration
11.58	278, 524	627	+	303	delphinidin 3,5-dihexoside	9.06
16.8	280, 524	448	+	287	cyanidin 3-glucoside	0.97
20.6	278, 524	640	+	317	petunidin 3,5-dihexoside	0.82
27.8	276, 524	462	+	301	peonidin 3-glucoside	0.14
30.3	280, 524	654	+	331	malvidin 3,5-dihexoside	3.26
31.7	278, 524	464	+	303	delphinidin 3-glucoside	3.26
34.5	280, 525	611	+	303	delphinidin 3-rutinoside	2.87
39.3	274, 530	492	+	331	malvidin 3-glucoside	1.46
30.1	298, 355	481/479	−	319/317	myricetin 3-glucoside	0.16
34	296, 356	627/625	−	481/319	myricetin 3-rutinoside	0.23
36.8	298, 350	609/610	−	301	quercetin 3-rutinoside	1.17
39.8	296, 352	463/464	−	301	quercetin 3-glucoside	1.47
40.5	300, 324	515	−	353.19	quercetin 3-galactoside	0.34
46.3	284, 352	447	−	301	quercetin 3-o-rhamnoside	1.02
46.9	264, 350	477	−	315	isorhamnetin 3-o-hexoside	0.74
48.1	266, 354	623	−	315	isorhamnetin3-o-hexoside-derivative	0.78
					Total	27.74

M^+^ is observed in positive mode. M^−^ is observed in negative mode. These measurements correspond to calafate fruit (DW). The values are expressed in mg g^−1^.

**Table 3:** Evaluation of the stability of anthocyanins encapsulated by spray drying over 336 days of storage at 5 °C.

Anthocyanin	Time of Storage	(mg g^−1^)
peonidin 3-glucoside	0	0.269 ^a^
petunidin 3,5-dihexoside	0	0.877 ^abc^
malvidin 3,5-dihexoside	0	0.987 ^abc^
cyanidin 3-glucoside	0	1.322 ^abc^
delphinidin 3,5-dihexoside	0	9.467 ^e^
peonidin 3-glucoside	24	0.316 ^a^
petunidin 3,5-dihexoside	24	0.895 ^abc^
cyanidin 3-glucoside	24	1.279 ^abc^
malvidin 3,5-dihexoside	24	1.550 ^bc^
delphinidin 3,5-dihexoside	24	9.600 ^e^
peonidin 3-glucoside	48	0.332 ^ab^
petunidin 3,5-dihexoside	48	0.791 ^abc^
malvidin 3,5-dihexoside	48	0.802 ^abc^
cyanidin 3-glucoside	48	1.800 ^c^
delphinidin 3,5-dihexoside	48	8.366 ^de^
peonidin 3-glucoside	168	0.302 ^ab^
cyanidin 3-glucoside	168	1.016 ^abc^
malvidin 3,5-dihexoside	168	1.111 ^abc^
petunidin 3,5-dihexoside	168	1.123 ^abc^
delphinidin 3,5-dihexoside	168	7.911 ^d^
peonidin 3-glucoside	336	0.151 ^a^
malvidin 3,5-dihexoside	336	0.569 ^abc^
cyanidin 3-glucoside	336	0.731 ^abc^
petunidin 3,5-dihexoside	336	0.941 ^abc^
delphinidin 3,5-dihexoside	336	7.439 ^d^

Different superscript letters in the same row mean significant differences at (*p* ≤ 0.05).

## Data Availability

Data is contained within the manuscript and in Supplementary Data.

## Supplementary Materials

The following are available online at https://www.mdpi.com/article/10.3390/antiox10111830/s1, Figure S1: Electronic microscopy of Aβ_1-40_ Peptides (a) uncombined and (b) after 4 h of aggregation.
