# Supplementary figures and images for: Phenolic Compounds in Calafate Berries Encapsulated by Spray Drying: Neuroprotection Potential into the Ingredient
Source: Antioxidants (Basel). 2021 Nov 18;10(11):1830. doi: 10.3390/antiox10111830 (PMC8614940; doi:10.3390/antiox10111830)

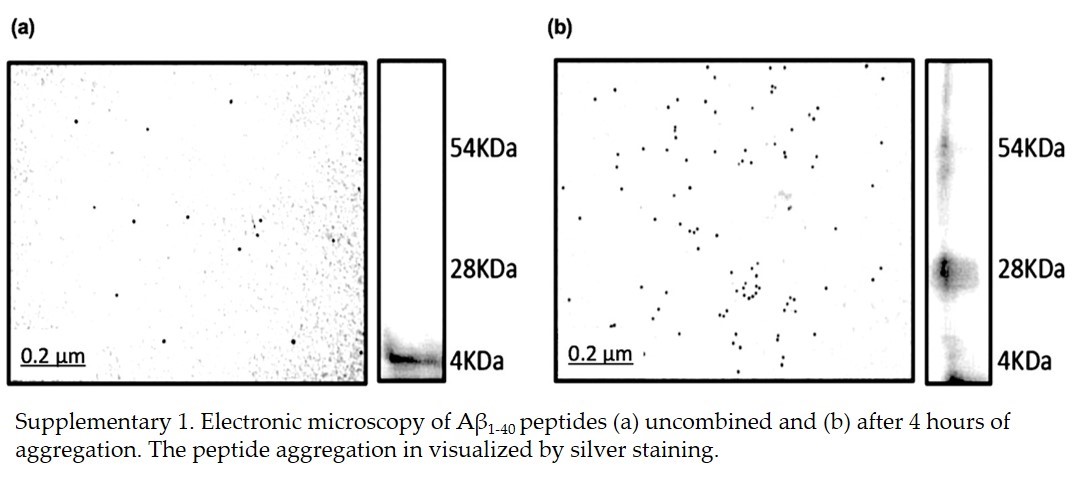

Supplement: Supplementary file 1 [file antioxidants-10-01830-s001.zip › antioxidants-1449263-supplementary.jpg]
